# Hospital outcomes of community-acquired COVID-19 versus influenza: Insights from the Swiss hospital-based surveillance of influenza and COVID-19

**DOI:** 10.2807/1560-7917.ES.2022.27.1.2001848

**Published:** 2022-01-06

**Authors:** Georg Marcus Fröhlich, Marlieke E. A. De Kraker, Mohamed Abbas, Olivia Keiser, Amaury Thiabaud, Maroussia Roelens, Alexia Cusini, Domenica Flury, Peter W. Schreiber, Michael Buettcher, Natascia Corti, Danielle Vuichard-Gysin, Nicolas Troillet, Julien Sauser, Roman Gaudenz, Lauro Damonti, Carlo Balmelli, Anne Iten, Andreas Widmer, Stephan Harbarth, Rami Sommerstein

**Affiliations:** 1 HeartClinic Lucerne, Lucerne, Switzerland; 2 Charité – Universitätsmedizin Berlin, Berlin, Germany; 3 Infection Control program, Geneva University Hospitals and Faculty of Medicine, Geneva, Switzerland; 4 Institute of Global Health of the University of Geneva, Geneva, Switzerland; 5 Kantonsspital Graubünden, Department for General Medicine, Chur, Switzerland; 6 Kantonsspital St. Gallen, St. Gallen, Switzerland; 7 University Hospital Zurich, Division of Infectious Diseases and Hospital Epidemiology, and University of Zurich, Zurich, Switzerland; 8 Cantonal Hospital Lucerne, Children’s Hospital, Pediatric Infectious Diseases, Lucerne, Switzerland; 9 Department for General Medicine, Klinik Hirslanden, Zürich, Switzerland; 10 Department for General Medicine, Spital Thurgau, Frauenfeld, Switzerland; 11 Swissnoso, the National Center for Infection Control, Bern, Switzerland; 12 Department of Infectious Diseases, Central Institute, Valais Hospital, Sion, Switzerland; 13 Department for General Medicine, Kantonsspital Nidwalden, Stans, Switzerland; 14 Department of Infectious Diseases, Bern University Hospital, Bern, Switzerland; 15 Ente Ospedaliero Cantonale Ticino, Division of Infection control and Hospital Epidemiology, Bellinzona, Switzerland; 16 Department for Infectious Diseases, University Hospital Basel, Basel, Switzerland; 17 Department of Health Sciences and Medicine, Clinic St. Anna, University of Lucerne, Lucerne, Switzerland

**Keywords:** COVID-19, Influenza, surveillance, mortality, ICU, Switzerland, air-borne infections, viral infections, influenza, severe acute respiratory syndrome – SARS, influenza virus, clinic, epidemiology

## Abstract

**Background:**

Since the onset of the COVID-19 pandemic, the disease has frequently been compared with seasonal influenza, but this comparison is based on little empirical data.

**Aim:**

This study compares in-hospital outcomes for patients with community-acquired COVID-19 and patients with community-acquired influenza in Switzerland.

**Methods:**

This retrospective multi-centre cohort study includes patients > 18 years admitted for COVID-19 or influenza A/B infection determined by RT-PCR. Primary and secondary outcomes were in-hospital mortality and intensive care unit (ICU) admission for patients with COVID-19 or influenza. We used Cox regression (cause-specific and Fine-Gray subdistribution hazard models) to account for time-dependency and competing events with inverse probability weighting to adjust for confounders.

**Results:**

In 2020, 2,843 patients with COVID-19 from 14 centres were included. Between 2018 and 2020, 1,381 patients with influenza from seven centres were included; 1,722 (61%) of the patients with COVID-19 and 666 (48%) of the patients with influenza were male (p < 0.001). The patients with COVID-19 were younger (median 67 years; interquartile range (IQR): 54–78) than the patients with influenza (median 74 years; IQR: 61–84) (p < 0.001). A larger percentage of patients with COVID-19 (12.8%) than patients with influenza (4.4%) died in hospital (p < 0.001). The final adjusted subdistribution hazard ratio for mortality was 3.01 (95% CI: 2.22–4.09; p < 0.001) for COVID-19 compared with influenza and 2.44 (95% CI: 2.00–3.00, p < 0.001) for ICU admission.

**Conclusion:**

Community-acquired COVID-19 was associated with worse outcomes compared with community-acquired influenza, as the hazards of ICU admission and in-hospital death were about two-fold to three-fold higher.

## Introduction

During the first wave, the clinical impact of the COVID-19 pandemic was compared with seasonal influenza [1,2]. This comparison may have been because of its worldwide spread via respiratory particles and similar initial clinical presentation [3,4]. For both infections, the most common symptoms are cough, fever, dyspnoea or headache. However, COVID-19 appears more deleterious than influenza [5] as intensive care units (ICUs) were stressed, elective procedures were reduced, and national lockdowns resulted in considerable economic hardship for parts of the population.

During the influenza season in Switzerland, between 1,000 and 5,000 patients with influenza A/B need hospitalisation and up to 1,500 patients die each year [6]. In comparison, COVID-19 in Switzerland has already caused more than 704,000 infections, more than 29,000 hospitalisations, and more than 10,500 deaths as of 28 July 2021 [7,8].

Although influenza is a well investigated disease with clear preventive and treatment strategies, including vaccination and anti-viral drugs, these strategies were not fully defined for COVID-19 at the time of writing. Several treatment strategies (e.g., dexamethasone and remdesivir) are already in use or are in the process of being approved [9-11]. However, vaccination appears to be the most efficacious preventive strategy with a conferred 95% protection against the wildtype virus [12].

So far, only a few retrospective studies have directly compared hospital outcomes of COVID-19 and influenza [13-16]. Two of these studies have demonstrated that comorbidities can affect the course of COVID-19 and influenza [13,14]. The comparison of COVID-19 and seasonal influenza is important from a public health perspective, as COVID-19 challenges the healthcare system in a different way. Therefore, we investigated the differences in mortality and ICU admission among patients hospitalised for COVID-19 and influenza in Switzerland.

## Methods

### Study population, data sources and variables

This retrospective multi-centre cohort study included data from patients admitted to one of 14 hospitals in Switzerland, including all five Swiss university hospitals. Seven centres continuously collected data for all adult patients who were hospitalised for influenza A/B, including three university hospitals (start date: 28 October 2018). A total of 14 centres collected data for all adult patients who were hospitalised for COVID-19, including five overlapping centres with influenza and five university hospitals (start date: 19 February 2020).

All data were collected from the database *Hospital-based surveillance of COVID-19 and influenza cases in Switzerland.* This database was originally initiated to collect data for all hospitalised influenza cases during the influenza season 2018/19 by the Institute of Global Health at the University of Geneva and the Infection Control programme, Geneva University Hospital, Switzerland. A standardised, central REDCap-hosted electronic questionnaire for data collection was set up for data entry in the participating centres [17,18].

Hospital data included patient demographics, main comorbidities, non-invasive or invasive ventilation, cardiovascular, pulmonary, or neurologic complications, hospital and ICU admission and discharge date, as well as death [17,18]. Inclusion required a positive real-time polymerase chain reaction (RT-PCR) obtained from any respiratory sample.

Only data for completed hospitalisations of patients > 18 years up to 22 July 2020 were included in the analyses. Potential hospital-acquired infections (with RT-PCR diagnosis obtained later than 2 days after admission) were excluded, as we wanted to focus on patients with influenza or COVID-19 present on admission. Sampling and RT-PCR processing were performed according to the local centre’s protocols.

### Outcome measures

The primary outcome measure was defined as in-hospital all-cause mortality. Secondary outcome was admission to the ICU. Further exploratory end-points included cardiovascular, pulmonary, renal or neurologic complications during hospitalisation or length of stay and antibiotic treatment.

### Statistical analysis

Pearson’s chi-squared test was used for categorical data and Mann-Whitney U test was used for ordinal data. Missing data were excluded for the calculation of the rate and the comparative tests.

For the primary and secondary outcome analyses, we used cause specific Cox hazard models to account for competing risks (hospital discharge for hospital mortality and discharge and death before ICU admission for ICU admission) to prevent overestimation of the impact of COVID-19 [19]. Patients who stayed more than 30 days were right-censored in all models. To adjust for differences in baseline characteristics, inverse probability weighting was applied using clinical and epidemiological factors present at admission significantly associated with influenza and COVID-19 as determined by univariate logistic regression models. We applied truncation at 1^st^/99^th^ percentile. In a second step, subdistribution hazard analysis using the Fine-Gray model was applied to determine the cumulative risk of death or ICU admission significantly associated with COVID-19 or influenza [20]. The main analyses were adjusted for sex, age and university hospital as treatment centre (available for all cases).

A more detailed description of the methods and of additional analysis is provided in the Supplementary Materials. As the date of outcome was only recorded for mortality and ICU admission, we calculated the crude incidence for all other outcomes.

All analyses were performed in R, Version 4.02 (R Foundation, Vienna, Austria) using the packages *survival, ipw, CausalGAM* and *cmprsk*. Two-tailed tests were performed and p values < 0.05 were considered statistically significant.

### Ethical statement

The study was approved by the Ethics Committee of the Canton of Geneva, Switzerland (CCER 2018–00577, 2020–00827). Data collection was also approved by all local ethics committees. The planning conduct and reporting of studies was in line with the Declaration of Helsinki, as revised in 2013.

## Results

### Baseline characteristics

In total, 4,441 patients were available from the two surveillance databases, of which 217 patients were excluded (170 for COVID-19 and 47 for influenza) because hospital admission or discharge date were missing.

This study includes 2,843 patients from 14 acute care hospitals in Switzerland with a confirmed diagnosis of community-acquired COVID-19 hospitalised between 19 February 2020 and 22 July 2020. This represents 50% of all reported hospitalisations in Switzerland during this period [21] (4,569 hospitalisations as of 3 September 2020). In addition, it includes 1,331 patients with community-acquired influenza A (96.4%) and 50 patients (3.6%) with community-acquired influenza B from seven centres in Switzerland hospitalised between 26 October 2018 and 26 March 2020. The majority of patients with COVID-19 (2,476/2,843, 87%) as well as those with influenza A/B (1,220/1,381, 97%) were directly referred to one of the participating hospitals. Several patients were transferred from local district hospitals to specialised facilities (4.9%, n = 139) of COVID-19 patients and 3.3% (n = 46) of influenza patients). In addition, 4.8% (n = 137) of the patients with COVID-19 and 5.2% (n = 72) of the patients with influenza were transferred from a long-term care facility (LTCF) .

Patients with COVID-19 were younger (median 67 years; interquartile range (IQR): 54–78) than patients with influenza A/B (median 74 years; IQR: 61–84; p < 0.001) and a greater percentage were male (61%) than female (48%) (p < 0.001) (Table 1).

**Table 1:** Baseline characteristics of patients with COVID-19 (19 February – 22 July 2020) and influenza A/B (26 October 2018 – 26 March 2020) in the Swiss surveillance database, Switzerland (n = 4,224)

Characteristics	SARS-CoV-2 (n = 2,843)		Influenza A/B (n = 1,381)		p value
Age (years), median (IQR)	67 (54–78)		74 (61–84)		< 0.001
Sex	n	%	n	%	
Female	1,121	39.4	715	51.8	< 0.001
Admission to university hospital	n	%	n	%	
Admissions	1,597	56.2	935	67.7	< 0.001
BMI (kg/m^2^), median (IQR)	26.7 (23.7, 30.5)^a^		25.2 (21.8, 29.4)^b^		< 0.001
Comorbidities	n	%	n	%	
Diabetes mellitus	522	26.9^c^	291	28.2^d^	0.578
Chronic cardiovascular disease	737	38.0^c^	473	45.9^d^	< 0.001
Renal impairment	374	19.3^c^	243	23.6^d^	0.011
Chronic pulmonary disease	413	21.3^c^	322	31.2^d^	< 0.001
Chronic neurologic impairment	276	14.2^c^	190	18.4^d^	0.002
Haematological disorder	39	2.0^c^	160	15.5^d^	< 0.001
Chronic liver disease	114	5.9^c^	57	5.5^d^	0.873

BMI: body mass index; COVID-19: coronavirus disease; IQR: interquartile range; SARS-CoV-2: severe acute respiratory syndrome coronavirus 2.

^a^ Missing values n (%): 856 (30%) as reporting of BMI/comorbidities was not mandatory and omitted by some centres.

^b^ Missing values n (%): 518 (38%) as reporting of BMI/comorbidities was not mandatory and omitted by some centres.

^c^ Missing values n (%): 904 (32%) as reporting of BMI/comorbidities was not mandatory and omitted by some centres.

^d^ Missing values n (%): 350 (25%) as reporting of BMI/comorbidities was not mandatory and omitted by some centres.

### Outcomes

Table 2 provides an overview of the crude clinical outcomes for patients with COVID-19 and patients with influenza A/B. Crude hospital mortality among patients with COVID-19 was almost three times higher than for influenza A/B patients (12.8% for patients with COVID-19 and 4.4% for patients with influenza A/B). ICU admission was also more frequent among patients with COVID-19 (19.4%) compared with patients with influenza (9.8%). Similarly, invasive ventilation was more common in patients with COVID-19 (13.9%) compared to patients with influenza (4.8%) (p < 0.001). The crude overall length of hospital stay was 8 days for COVID (IQR: 5–16 days) and 7 days for influenza (IQR: 7–13) (p < 0.001).

**Table 2:** Unadjusted crude outcomes for patients with microbiologically confirmed community-acquired COVID-19 (19 February – 22 July 2020) or influenza A/B infections (26 October 2018 – 26 March 2020), Switzerland (n = 4,224)

	SARS-CoV-2 (n = 2,843)		Influenza A/B (n = 1,381)		p value
	n	%	n	%	
In-hospital deaths	363	12.8	61	4.4	< 0.001
Admission to the ICU	483	19.4	129	9.8	< 0.001
Invasive ventilation	394	13.9	66	4.8	< 0.001
Respiratory complications	2,082	93.6^a^	744	83.9^b^	< 0.001
Cardiac diseases	556	25.0^a^	212	23.9^b^	0.291
Neurologic impairment	394	17.7^a^	82	9.2^b^	< 0.001
Renal impairment	601	27.0^a^	149	16.8^b^	< 0.001
Length of hospital stay, median (IQR)	8 (5–16)		7 (4–13)		< 0.001
Any antibiotic treatment	1,539	61.7^c^	862	65.7^d^	< 0.001

COVID-19: coronavirus disease; ICU: intensive care unit; IQR: interquartile range; SARS-CoV-2: severe acute respiratory syndrome coronavirus 2.

^a^ Missing values n (%): 618 (22%) as reporting of these complications was not mandatory and omitted by some centres.

^b^ Missing values n (%): 494 (36%) as reporting of these complications was not mandatory and omitted by some centres.

^c^ Missing values n (%): 349 (12) as reporting of these complications was not mandatory and omitted by some centres.

^d^ Missing values n (%): 69 (5) as reporting of these complications was not mandatory and omitted by some centres.

**Figure 1:**
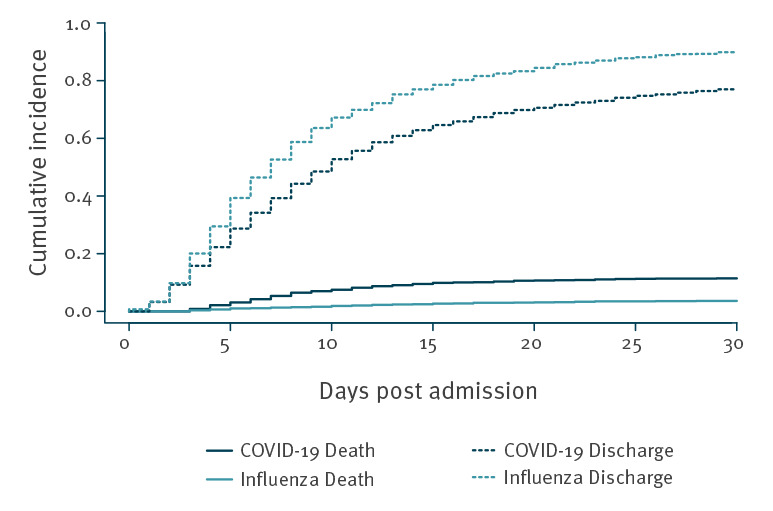
Cumulative incidence plot of mortality with discharge as competing risk by disease status, COVID-19 (19 February–22 July 2020) versus influenza (26 October 2018–26 March 2020), Switzerland (n = 4,224)

The adjusted subdistribution hazard ratio (sdHR) for in-hospital mortality was 3.01 (95% CI: 2.22–4.09; p < 0.001) for COVID-19 compared with influenza and 2.44 (95% CI: 2.00–3.00; p < 0.001) for ICU admission (selected variables and balance of weights in Supplementary Table 1). The corresponding cumulative incidence plots for death and the competing risk discharge is shown in Figure 1. The direct effect of COVID-19 on in-hospital death had a cause-specific hazard ratio (csHR) of 2.58 (95% CI: 1.90–3.50; p < 0.001), and the csHR for hospital discharge was 0.78 (95% CI: 0.72–0.84; p < 0.001). That is, patients with COVID-19 stayed in hospital longer than influenza patients, which indirectly increased their risk of in-hospital death.

COVID-19: coronavirus disease.

The largest increase in cumulative incidence of COVID-19 deaths occurred from day 5 (2.1%) to day 15 (9.9%), whereas the increase in cumulative incidence of influenza deaths was 0.7% and 2.7%, respectively. The cumulative incidence for influenza discharge was 29.4% on day 5 and 78.6% on day 15, whereas the cumulative incidence for COVID-19 discharge on day 5 and day 15 was 22.2% and 64.6%, respectively.

**Figure 2:**
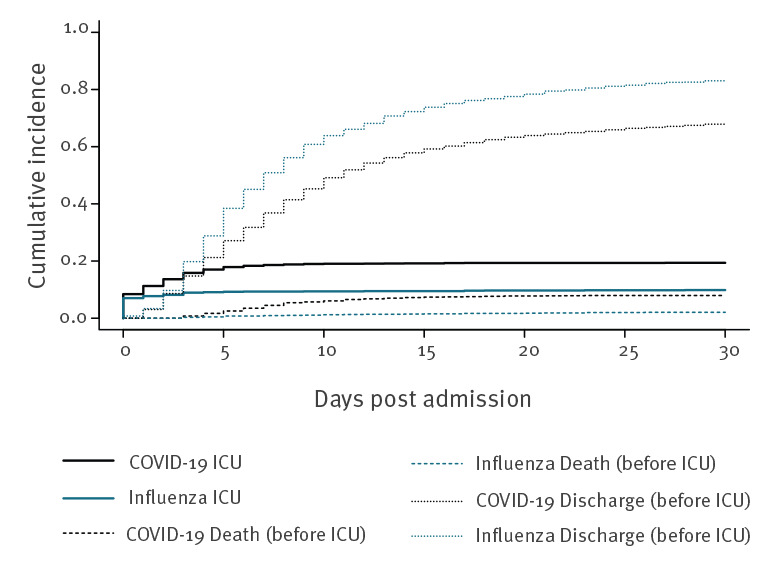
Cumulative incidence plot of ICU admission with discharge and death before ICU admission as competing risk by disease status, COVID-19 (19 February–22 July 2020) versus influenza (26 October 2018–26 March 2020), Switzerland (n = 4,224)

COVID-19: coronavirus disease; ICU: intensive care unit.

For the secondary outcome COVID-19-associated ICU admissions (Figure 2), the direct effect of COVID-19 had a csHR of 2.46 (95% CI: 2.00–3.01; p < 001). Indirectly, COVID-19 altered the risk of ICU admission because patients with COVID-19 died earlier during their stay (csHR for death before ICU admission: 3.93; 95% CI: 2.61–5.95; p < 0.001). The csHR for the competing risk of being discharged before ICU admission was not significantly associated with COVID-19 compared to influenza (0.95; 95% CI: 0.87–1.03; p = 0.181).

Of note, the cumulative incidence of ICU admission, considering competing events, increased from 8.4% to 17.9% over the first 5 days for COVID-19, but it increased only from 7.0% to 9.2% for influenza over the same period. The results of the weighted csHR are summarised in Supplementary Table 3.

### Sensitivity analysis

Of the 2,634 complete cases, the adjusted sdHR for mortality was slightly higher at 3.14 (95% CI: 2.11–4.68; p < 0.001). The balance of weights for confounders are shown in Supplementary Table 2. The adjusted sdHR for mortality in the subgroup of 4,232 cases (excluding 209 direct transfers from LTCF) was 3.10 (95% CI: 2.23–4.32; p < 0.001), and the adjusted sdHR for mortality in the subgroup of 4,391 Influenza A and COVID-19 cases (excluding 50 with Influenza B) was 2.87 (95% CI: 2.11–3.90; p < 0.001). Finally, the adjusted sdHR for ICU admission in 4,139 cases, excluding the 302 patients directly admitted to the ICU on the day of hospitalisation, was higher at 5.05 (95% CI: 3.54–7.20; p < 0.001). The cumulative incidence plot is shown in Supplementary Figure 1.

## Discussion

This study directly compares the outcomes of patients hospitalised for influenza A/B or COVID-19 in a nation-wide Swiss surveillance database. After considering competing events as well as imbalances between patient groups, we found that community-acquired COVID-19 was associated with a threefold increase in the cumulative risk of in-hospital death compared with influenza A/B. This increase was associated with a higher daily mortality risk as well as an increased length of hospital stay for patients with COVID-19. Similarly, patients with COVID-19 had a more than two-fold increased risk of being transferred to the ICU than influenza patients.

These numbers are in line with a large French study by Piroth et al., where the in-hospital mortality rate was 16.9% for COVID-19 and 5.8% for influenza. Similarly, they reported that the need for ICU admission was higher in patients with COVID-19 (16.3%) than in patients with influenza (10.8%) [15].

The COVID-19 pandemic poses a tremendous medical and socioeconomic burden for many countries. Early in the pandemic, however, COVID-19 was downplayed in some communications as only a ‘little flu’ [1,22,23]. Now we know that the characteristics as well as the course of COVID-19 appear to be more serious than for influenza in several aspects. Severe acute respiratory syndrome coronavirus 2 (SARS-CoV-2) and in particular several of its variants appear to be more contagious than the influenza virus, with a reproductive number of 2–2.5, and contagious even in patients without symptoms or before symptoms appear [3]. Moreover, according to the World Health Organization (WHO), in unvaccinated populations 15% of COVID-19 cases might need hospitalisation and 5% of hospitalised patients require ventilation [3]. In comparison, a study from Germany of 34,493 patients with influenza found that 3.7% of these patients needed hospitalisation, and 5% of these received invasive ventilation [24]. In our study, 13.9% of the hospitalised patients with COVID-19 needed mechanical ventilation, compared to 4.7% of the hospitalised patients with influenza. These numbers underline the clinical importance of COVID-19.

In our study, patients with COVID-19 were predominantly male with a median age of 67 years, and patients with influenza were predominantly female with a median age of 74 years. Therefore, the years of potential life lost are likely to be higher in COVID-19. This hypothesis is supported by the finding that the patients with COVID-19 were less likely to have some of the comorbidities than patients with influenza. However, all the clinical outcomes for patients with COVID-19 were worse than those for patients with influenza and persisted even after adjustment for possible confounders. The WHO estimates case fatality rates of 3–4% for COVID-19, whereas the infection fatality rates for seasonal influenza are usually much lower, around 0.1% [2]. In our Swiss hospital surveillance, the cumulative incidence of mortality of hospitalised patients with COVID-19 up to day 30 was 11.4% compared with 3.6% in hospitalised influenza patients. Previous studies reported mortality rates of hospitalised patients with COVID-19 between 4% and 28% [25,26].

In the presented Swiss cohort, the cumulative incidence of patients with COVID-19 with ICU admission up to day 30 was 19.4% compared with 9.9% among influenza patients. Interestingly, patients with influenza who needed intensive care were transferred to ICU soon after hospital admission. In contrast, the proportion of ICU transfers for patients with COVID-19 increased steadily up to 5 days after hospital admission.

Apart from respiratory complications, neurologic impairment or kidney failure were also more common among the COVID-19 patients. It is unclear whether this finding might be explained by vasculitis and thromboembolic events, which are common in COVID-19 [27]. Surprisingly, there was no difference in cardiovascular complications. Although antibiotic therapy was more common in patients with influenza in this Swiss cohort, it remains unclear whether influenza is associated with pulmonary bacterial superinfection more often than COVID-19.

The present study has several limitations. Influenza seasons may vary considerably in their severity depending on circulating viruses. In the presented analysis, only data from two seasons – 2018/19 (n = 1,010) and 2019/20 (n = 371) – were available. In addition, detection bias of in-hospital influenza cases cannot fully be excluded. In this regard, a difference was noted between university hospitals and non-university hospitals: detection of influenza cases was statistically significant more frequently in university hospitals. This may hint at a need for more stringent case detection for COVID-19, especially in non-university hospitals. Therefore, some mild influenza cases may have been missed and this may have even led to an underestimation of the severity of COVID-19. Nevertheless, the main analysis was adjusted for type of hospital (university and other). Irrespective of hospital type, the results remained unchanged. Another possible bias is related to missing data. For about one-third of the patients in the database, comorbidity data were lacking because it was not mandatory for the surveillance. As missingness was centre-specific (missing at random) and only related to independent variables, we conducted complete case analyses for the primary outcome. The point-estimate of the analysis including all patients but excluding BMI and comorbidities from the propensity weights fell within the 95% CI of the main analysis, indicating that excluding incomplete cases did not introduce a major bias.

The impact of various treatment strategies could not be analysed in the presented Swiss cohort at the time of writing. It can be assumed that mortality for the patients with COVID-19 was relatively high, as the majority of the presented cases occurred early during the COVID-19 pandemic and knowledge about the best supportive treatment strategies has slowly increased. As such, the current findings may not apply to future COVID-19 cases.

Data on type of COVID or influenza strains were not available, but influenza B cases were rare (only 50 patients), a finding that suggests that there was no substantial bias for grouping influenza A and B together.

Finally, since no data were available for co-infection of influenza virus and SARS-CoV-2, this was not considered. However, several case reports found that only 4% of patients with COVID-19 had a co-infection with influenza virus [28], and no difference in the course of disease was noted in comparison to SARS-CoV-2 infections alone [28,29].

## Conclusion

In this retrospective Swiss multi-centre hospital cohort study, COVID-19 was associated with a three-fold higher hazard for hospital mortality and a two-fold higher risk of ICU transfer compared with seasonal influenza A/B infection. These results have important implications for decisions about public health interventions in the current COVID-19 pandemic.

## Supplementary Data

592000 Supplementary Material
