# Supplementary material for: Hospital outcomes of community-acquired COVID-19 versus influenza: Insights from the Swiss hospital-based surveillance of influenza and COVID-19
Source: Euro Surveill. 2022 Jan 6;27(1):2001848. doi: 10.2807/1560-7917.ES.2022.27.1.2001848 (PMC8739338; doi:10.2807/1560-7917.ES.2022.27.1.2001848)

This supplementary material is hosted by *Eurosurveillance* as supporting information alongside the article ‘Hospital outcomes of community-acquired COVID-19 versus influenza: Insights from the Swiss hospital-based surveillance of influenza and COVID-19’, on behalf of the authors, who remain responsible for the accuracy and appropriateness of the content. The same standards for ethics, copyright, attributions and permissions as for the article apply. Supplements are not edited by *Eurosurveillance* and the journal is not responsible for the maintenance of any links or email addresses provided therein.

Statistics

The method and results of the validation of the weights is provided in Supplementary Table 1. In a second step, subdistribution hazard analysis using the Fine & Gray model were applied to determine the cumulative risk of death or ICU admission associated with COVID-19 versus influenza. The main analyses were adjusted for sex, age, and university hospital as treatment center (available for all cases), to adjust for unmeasured confounders between university vs non-university centers. In a subgroup analysis we adjusted in addition for body weight and the presence of comorbidities. Since this information was not systematically collected by all centers we included only patients/centers where this info was available (n=2634; 1731 for COVID-19 and 903 for Influenza). There, we also adjusted for weight and presence of comorbidities (cardiovascular, asthma, respiratory, hemato-immunological, renal and neurological; Suppl. Table 2). Further subgroup analysis focused on i) "in-hospital transfers" to the ICU, excluding direct admission to the ICU from the community and ii) community transfers, excluding patients admitted from long term care facilities (LTCF), as during the COVID-19 pandemic fewer LCTF cases may have been transferred to hospital to prevent overburdening of hospital capacities and iii) a comparison of Influenza A with Covid-19 (excluding the 50 Influenza B cases), the exclude potential confounding of co-circulating Influenza A and B strains.

Supplementary Table 1: Balance check of inverse probability weights

We used balance.IPW from the CausalGAM package (Ref R): This function calculates weighted means of covariates and then examines the differences in the weighted means across influenza and COVID-19 cases as a diagnostic for covariate balance for inverse probability weighting.

The standardized mean differences between all variables in the dataset are reported along with a z-statistics for these standardized differences, whereby z-statistics closer to 0 imply better univariate mean balance.

The columns are (from left to right) the observed mean of the covariate among the COVID-19 patients, the observed mean of the covariate among the influenza patients, the weighted mean of the covariate among the COVID-19 patients, the weighted mean of the covariate among the influenza patients, the weighted mean difference, and the z-statistic for the difference.

The formula (logistic regression with logit-link) used for calculation of weights was:

Virus_type ~ Age + Gender + Admission_to_University Hospital

	obs.mean.C	obs.mean.I	w.mean.C	w.mean.I	w.mean.diff	z
Age	65.11	70.41	66.76	66.43	0.33	2.252
Gender (% female)	0.39	0.52	0.44	0.45	-0.01	-3.717
Admission to University hospital (%)	0.56	0.68	0.60	0.60	-0.00	-0.689

Supplementary Table 2: Balance check of inverse probability weights for complete case analysis

The formula (logistic regression with logit-link) was:

Virus_type Age + Gender + Admission_to_University Hospital + weight + com_cardiovascular + comorbidity_asthma + comorbidity _neuro_impair + comorbidity _renal + comorbidity _hemato_immuno

	obs.mean.Covid-19	obs.mean. Influenza	w.mean. Covid-19	w.mean. Influenza	w.mean.diff	z
Age	68.957	73.196	70.788	70.667	0.122	0.587
Gender (female)	0.406	0.494	0.439	0.445	-0.006	-0.897
Admission to university hospital	0.515	0.681	0.573	0.577	-0.004	-0.724
Weight	79.253	73.37	77.023	77.457	-0.434	-1.21
Comorbidities (Yes/No)						
Cardiovascular	0.392	0.464	0.423	0.423	0	0.041
Asthma	0.091	0.134	0.108	0.109	-0.001	-0.239
Neurological impairments	0.139	0.171	0.149	0.16	-0.011	-3.1
Renal	0.201	0.252	0.228	0.223	0.005	0.793
Hematological/immunological	0.021	0.159	0.074	0.065	0.009	1.525

The table contains the same information as explained above (Supplementary Table 1).

Supplementary table 3. Adjusted hazard ratios of primary and secondary outcomes (COVID-19 vs. influenza) by weighted cause-specific (CS) Cox models. Patients with hospital duration >30 days were right-censored at day 30.

	**CS Hazard ratio**	**95% Confidence interval**	**p-value**
Primary outcome analysis			
Hospital mortality	2.58	1.90-3.50	<0.001
Hospital discharge	0.78	0.72-0.84	<0.001
Secondary outcome analysis			
ICU admission	2.46	2.00-3.01	<0.001
Death before ICU admission	3.93	2.61-5.95	<0.001
Discharge alive before ICU admission	0.95	0.87-1.03	0.181

Supplementary Figure 1

Cumulative incidence plot. ICU admission with discharge and death before ICU admission as competing risk, by disease status (COVID-19 versus influenza); Patients directly admitted to the ICU from the community were excluded for this subgroup analysis.

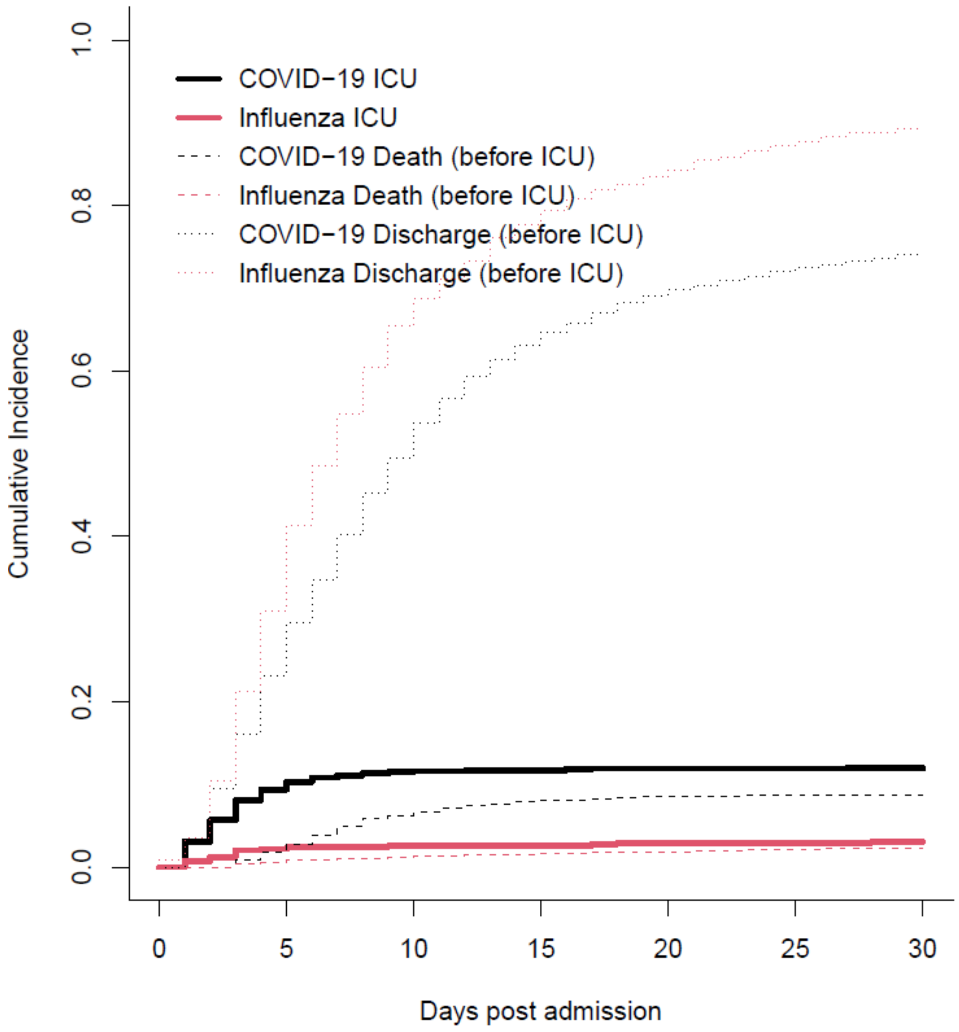

Supplement: Supplementary Material [file 20-01848_SOMMERSTEIN_Supplementary_material.pdf]
